# Manifolds in the Medial Premotor Cortex During Switching From Attending to Tapping to a Metronome

**DOI:** 10.1111/nyas.70259

**Published:** 2026-04-07

**Authors:** Dobromir Dotov, Abraham Betancourt, Jorge Gámez, Hugo Merchant

**Affiliations:** ^1^ Department of Biomechanics University of Nebraska Omaha Omaha Nebraska USA; ^2^ Departamento de Neurobiología del Desarrollo y Neurofisiología, Instituto de Neurobiología UNAM Campus Juriquilla Querétaro México

**Keywords:** beat perception and synchronization, manifolds, medial premotor areas, monkey neurophysiology, neural trajectories, rhythm‐based timing

## Abstract

Animals synchronize their movements with external rhythms to coordinate perception and action, but the neural population mechanisms that allow them to attend and then initiate and sustain these rhythms remain unclear. Using recordings from the medial premotor cortex (MPC) of two macaque monkeys, we investigated neural dynamics during an attend‐then‐synchronize tapping task with visual metronomes. We found low‐dimensional neural manifolds that captured neural population trajectories. During the attention epoch, trajectories exhibited increasing amplitude and oscillatory strength with the successive stimuli, which is consistent with a dynamic system using a resonant mechanism. Then, the transition from perception to tapping synchronization was marked by a reliable shift into a distinct manifold subspace with rotatory dynamics, enabling accurate decoding of the switch in behavior. In addition, correct tapping trials were characterized by a more robust oscillatory structure not seen in incorrect trials. These findings demonstrate that the geometry and coherence of MPC neural trajectories encode different perceptual and motor aspects of tapping synchronization.

## Graphical abstract

Low‐dimensional manifolds emerged in neural populations of the medial premotor cortex of macaque monkeys while they performed an attend‐then‐synchronize tapping task with periodic visual stimuli. The neural dynamics followed an increasingly oscillatory trajectory from attending to tapping stages of the trial (left panel). The trajectory moved into a different region from attending to tapping, as shown by accurate behavioral state decoding with a classifier (right panel).

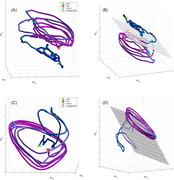

## 1 Introduction

Animals coordinate their actions with predictable events in their surrounding environment to perceive, move, detect danger, and communicate with each other [1, 2, 3]. For communication, different forms of rhythm exist in different species as a function of their vocalization capacities and ecological demands [4, 5, 6, 7]. Group synchronization can confer a stability advantage to the rhythmic performance of individuals [8]. Notably, beat‐based rhythm in music and dance affords synchronous interactions between participants [9] and engages sensorimotor synchronization that is linked with complex loops of attention, perception, and action [10, 11, 12]. Beat perception allows predicting or anticipating future events [13, 14] by co‐opting the motor system [15, 16, 17, 18]. The human audiomotor system is designed to extract the temporal patterns from continuous streams of sounds and to perceive a steady pulse or beat in music spontaneously [19]. The beat is a periodic internal representation that is projected into the future to predict when the next event is likely to occur and to trigger the initiation of movements so that they coincide or anticipate this cognitive event [20]. Crucially, there is evidence that an internal or covert beat is present in a variety of rhythmic tasks [21, 22, 23, 24]. In perceptual tasks, it can be detected in large‐scale brain activity as EEG oscillations of prominent amplitudes at the frequencies of the beat [25, 26]. This is the case for both weakly and strongly periodic auditory stimuli [21, 27]. How beat‐based rhythm can harness populations of neurons is still debated, and several explanatory mechanisms have been proposed. In this work, we first briefly review these mechanisms and then use high‐density neural recordings from macaques to investigate how population dynamics emerge while attending and tapping along an isochronous metronome.

### 1.1 Neural Entrainment and Neural Resonance

**FIGURE 1:**
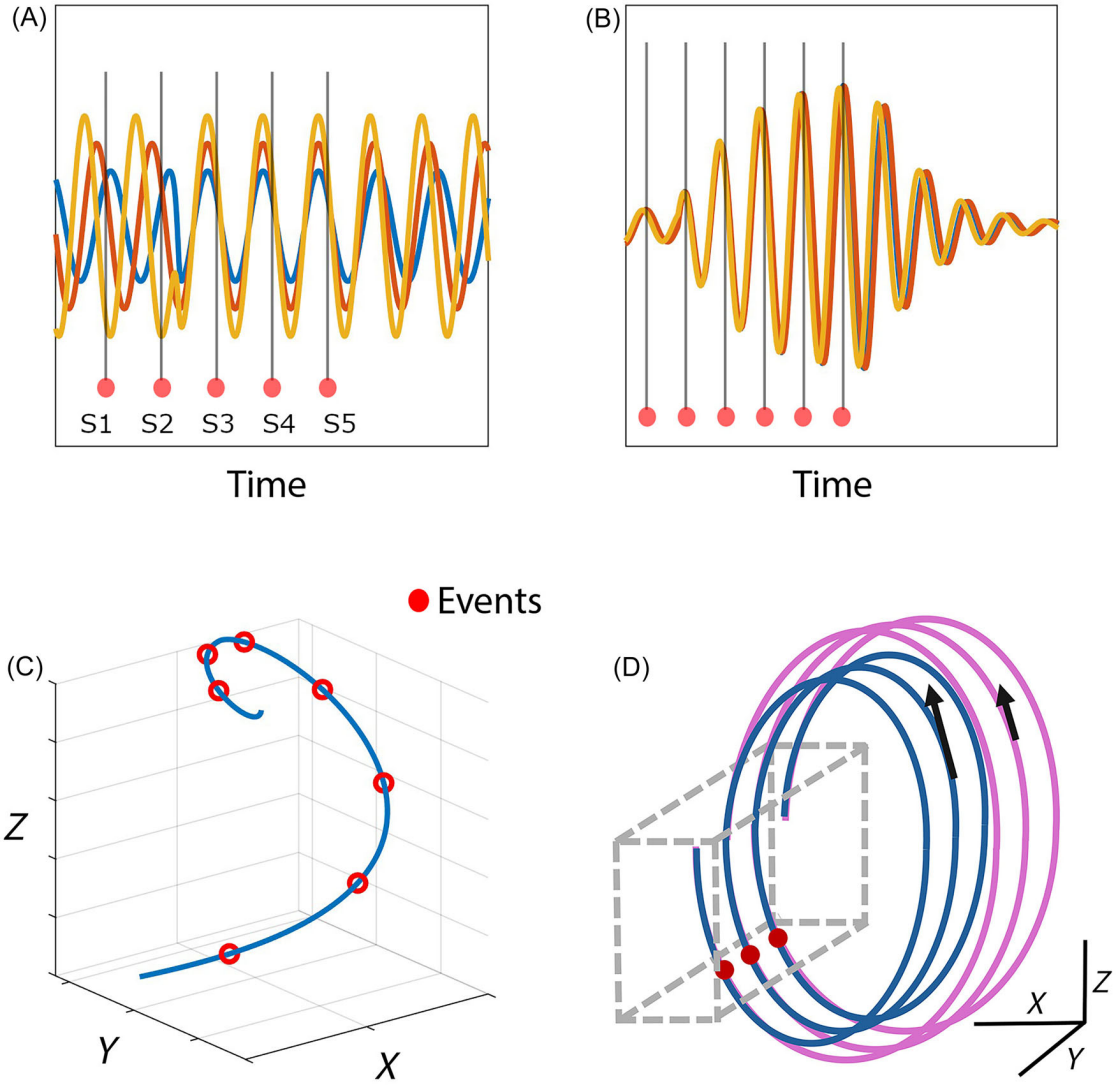
Schematic illustrations of different but not mutually exclusive mechanisms for organizing neural populations during synchronization with rhythmic events. For the sake of generality, events marked as red dots could be either stimuli or motor events. (A) Entrainment is characterized by the alignment of intrinsic ongoing oscillations, phase‐shifted by periodic events (S1–S5), represented by the vertical lines. The intrinsic oscillations change phase until they are phase‐locked by S5. (B) Resonance is characterized by emerging oscillations with increasing amplitude when the events have a frequency close to a preferred frequency of the neural dynamics. The intrinsic oscillations increase in amplitude and reach a maximum by S5. (C) A low‐dimensional neural state trajectory unfolds along the duration of the trial. Presumably, the dimensions *X*, *Y*, and *Z* are on a task‐related manifold that is embedded inside the high‐dimensional space of neuronal firing rates. (D) Similar to (C), low‐dimensional neural state trajectories during a rhythmic tapping task with either of two different intervals, shown in blue (short) and pink (long). The amplitude and speed (tangent vectors) are candidate parameters for encoding the interval duration [43, 44]. Tap times are found within a convergent section of the state space, shown as a dashed box.

One of the influential models in this context, neural entrainment, studies how an external rhythm can phase‐synchronize intrinsic brain oscillations and focus sensory processing of stimuli that occurs on the beat [28, 29]. This shares certain features with dynamic attending theory, according to which the synchronization of neural oscillations to external stimuli, such as speech and music, creates a rhythm of attention with enhanced and suppressed processing at salient points [30]. Entrainment models can be characterized specifically by the implication that intrinsic self‐sustained oscillations must preexist the stimulus so that they can be entrained by it [31] (see Figure 1A). Neural resonance is an alternative mechanism in which amplitude and oscillation dynamics are not intrinsic but emerge as a result of the rhythmic input of stimulus energy (Figure 1B) [32, 33, 34, 35]. Mathematical models show that populations of weakly coupled excitable units, driven by resonance phenomena, can exhibit time‐keeping properties [36, 37]. As a dynamic phenomenon, resonance occurs when a system is being forced at frequencies closely aligned with its internal timescales. In this regime, the energy of the forcing stimulus is not lost but retained, leading to an amplified response of the driven system. In nonlinear systems, resonant responses can also arise at different frequencies related to the forcing by an integer ratio. Neuronal circuits, through mechanisms such as synaptic plasticity, delay, and adaptation of natural frequencies, can instantiate dynamic systems that resonate selectively to rhythms in the sensory input [13].

### 1.2 Neurophysiology of Synchronization to a Metronome in the Rhesus Monkey

Questions about the nature of neural dynamics in rhythmic tasks can be addressed in detail by studying rhesus monkeys. Their rhythmic abilities and phylogenetic closeness to the human audiomotor system make them an interesting model species to trace the emergence of rhythmic skills [38]. For the past 20 years, our lab has been showing that macaques have all the necessary audiomotor machinery to perceive and synchronize to the beat [39]. EEG studies in the rhesus monkey have shown that macaques produce evoked potentials linked to the detection of isochronous auditory patterns [40], as well as to subjectively accented 1:2 and 1:3 rhythms from auditory metronomes [41, 42]. In addition, monkeys trained on tapping tasks can flexibly and predictively produce periodic intervals in synchrony with auditory and visual metronomes [43, 44] and can continue tapping isochronically without sensory cues [45, 46]. Notably, they tap consistently to the subjective beat of music excerpts with different tempos, choosing freely a tapping phase for each song [20]. Furthermore, monkeys show an error correction mechanism during synchronization that helps them control the duration of the produced intervals, which is more efficient at the preferred tempo of the animals [47]. Therefore, these observations suggest that during tapping synchronization, monkeys use a complex rhythmic timing mechanism that includes both attentional and predictive components.

Imaging studies in humans have shown that the neural substrate of beat‐based timing includes pathways within the voluntary motor control system, including medial premotor areas (medial premotor cortex [MPC]: supplementary motor area [SMA] and pre‐supplementary motor area [pre‐SMA]), basal ganglia (most often the putamen, but also the caudate nucleus and globus pallidus), and the motor thalamus, all of which form one of the cortico‐basal ganglia‐thalamocortical loops [48, 49, 50]. In the macaque, recent neurophysiological studies indicate that the MPC is involved in the internal pulse while tapping to a metronome [51, 52]. Cells in an MPC population are recruited in rapid succession, producing neuronal sequences with a progressive pattern of activation. This flexibly fills the beat duration and indicates relative interval progressions [53, 54].

### 1.3 Neural Population Trajectories

The advent of high‐density neural recordings using implanted electrodes in animal models has enabled a closer examination of the relationship between individual neurons and population dynamics in a given area of the cortex [55, 56, 57]. Populations of neurons combine sparse coding, where few neurons are strongly tuned to a given stimulus feature, and mixed selectivity, where neurons are tuned to multiple statistically independent behavioral parameters. As a result, the same population can respond flexibly to different stimuli [58]. Of interest to us, this suggests that we need to study how the same neural population responds to different intervals and different epochs of the task.

Central to the present work is the substantial interest in the fact that the many neurons recorded in a high‐density setup can embed a low‐dimensional state space when the individual cells are correlated. This is uncovered with dimensionality‐reduction techniques that take as input the high‐dimensional set of individual activities and extract a low‐dimensional set by projecting them on fewer variables and leaving out redundancies [59, 60]. The reduced variables define a neural state space, often characterized as a smooth manifold inside the original high‐dimensional space [61]. Neural manifolds contain the possible states of a population of neurons, given the combination of intrinsic constraints, such as anatomy and neurophysiology, and extrinsic constraints, in the form of behavioral dynamics [62]. The geometry and kinematics of the state trajectory through the manifold can encode behavioral events (see Figure 1C), and its parameters correspond to parameters such as interval duration [63, 64], reaching for a target [65, 66, 67], and switching between waiting and execution [68]. In premotor and motor areas, population dynamics compute preparation parameters and trigger targeted movements [69, 70].

### 1.4 Population Trajectories in Rhythmic Tasks

It is known that neural trajectories often exhibit a rotational component [68, 71]. Yet, most studies of state space manifolds have been done in the context of single‐shot behaviors, such as waiting and reaching, leaving fewer opportunities to study manifolds in rhythmic tasks. In simulations, it is possible to train recurrent neural networks to exhibit oscillations on a low‐dimensional reduced space [72, 73]. In the macaque's rhythmic tapping and synchronization with a stimulus, cyclic evolution is evident when the time‐varying activity of MPC neurons is projected onto a low‐dimensional state space. The population trajectories exhibit a list of interesting properties, summarized schematically in Figure 1D [74]. They have recurrent or regenerating loop dynamics for each produced interval; they converge to a narrow state space at the time of tapping, resetting the beat‐based clock at this point. This internal representation of rhythm could be transmitted as a phasic top‐down predictive signal to sensory areas, and geometric features such as speed and amplitude encode the produced interval duration. Furthermore, we showed that this neural population chronometer is bimodal, having the same dynamic properties in response to both auditory and visual metronomes [43].

This study investigates how said neural trajectories arise when a rhythmic stimulus is attended, even before tapping begins. To this end, we employed the same overall animal training and testing framework and data stream, but we focused on the neural trajectory before the onset of tapping. Specifically, we employed a task with two epochs: a perceptual epoch in which the monkeys attended to the metronome stimulus without moving, and a tapping epoch in which the monkeys synchronized their movements with the stimulus. The neural population trajectories in MPC showed two different state space manifolds, one for each task epoch. In addition, there was a gradual increase in their amplitude, and the robustness of their oscillation of each loop was linked to the sequence of produced intervals during tap synchronization to the metronome.

## 2 Methods

### 2.1 Subjects

Two monkeys (*Macaca mulatta* [M1, male, 10 kg, 10 years old; M2, female, 7 kg, 8 years old]) performed the experiments after extensive training. Monkeys were obtained from a specialized *M. mulatta* breeding company in Mexico City, called Proyecto Camina A.C., which follows international standards of reproduction and animal care. The company does not catch animals in the wild and has certified veterinary care.

All the animal care, housing, and experimental procedures were approved by the Ethics in Research Committee of the Instituto de Neurobiología UNAM, protocol 090.A, which follows the 3Rs, and conformed to the principles outlined in the Guide for the Care and Use of Laboratory Animals (NIH, publication number 85‐23, revised 1985).

The animals are housed in a monkey facility with cages of 1.5 m^3^, with controlled temperature (average of 22°C), humidity, and a 12/12‐h day/night cycle. In our Institute, we have three certified veterinarians who continuously provide care for the monkeys and perform regular health checkups and medical analysis of the animals. Animal care staff keep the facilities clean, and they provide food and water to the animals 365 days of the year. In addition, monkeys are monitored daily by researchers and the animal care staff to check their conditions of health and welfare.

The monkeys were fed ad libitum with a special diet of the brand LabDiet (Monkey Diet 5038) and had daily access to raisins and berries. Water was provided on correct trials during training and experimental sessions 5 days a week. Monkeys receive between 200 and 250 mL of water and/or juice per day during these days. If performance was especially poor or the monkey did not engage much with the task, additional water to complete the minimum 200 mL was provided through soaking dry food pellets in the required amount of water. Following the last session of the week, monkeys were given additional water up to 350 mL. The monkeys also received fruit such as apples and bananas after experimental sessions and on weekends.

### 2.2 Task

**FIGURE 2:**
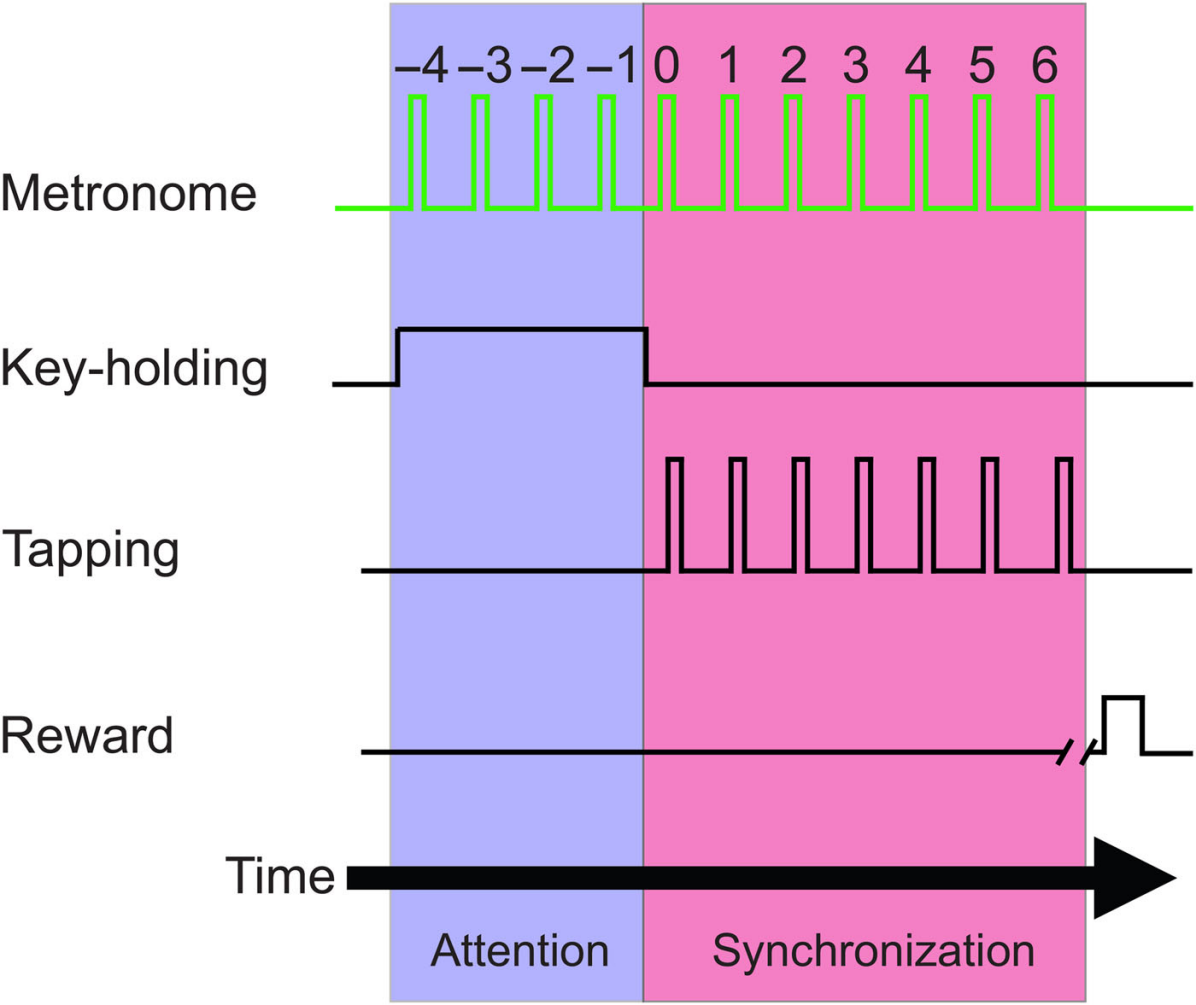
Schematic illustration of the task, which consisted of two stages. In the first stage, the animal watched the stimulus (attention) while attending to the three intervals of the metronome. The animal maintained her responding hand on a key. Then, in the second stage, the animal started tapping a button in synchrony with the metronome for six intervals to obtain a reward (synchronization).

Unlike one‐shot actions, such as reaching and single‐interval timing, rhythmic tapping presents the unique opportunity to measure parameters of the oscillatory dynamics of the neural trajectory. The monkeys were trained to perform an attend‐then‐synchronize‐task (AST) [44], which is used to investigate neural population dynamics during rhythmic tasks [43, 74]. The animals attended to and then tapped in synchrony with a sequence of discrete periodic visual stimuli (see Figure 2).

A trial consisted of a sequence of between nine and 11 events with a fixed interstimulus interval (ISI). Trials were separated by a random intertrial interval in the range 1.2–4 s. At the beginning of a trial, the animals had to hold a lever and withhold moving for at least three intervals before they could begin tapping seven times in response to the metronome. Trials were terminated if the animal started tapping too early, too late, or with a large asynchrony. The reward, fruit juice, was given when the duration of the produced intervals showed an error below 18% of the stimulus interval and all asynchronies between stimuli and taps were less than ±200 ms. ISI duration was a trial condition, with six randomly presented tempos ranging from 450 to 950 ms. M1 performed 1941 trials, 38% correct. M2 performed 2151 trials, 72% correct.

In each data collection session, the monkey was seated and restrained in a primate chair in a sound‐attenuated room. The animal tapped on a push button with one hand while the opposite arm was comfortably restrained. The target event was a red square with a side length of 5 cm, presented for 33 ms on a 17ʺ CRT monitor, 60 Hz refresh rate, at 60 cm from the animal. Stimulus presentation was controlled by a Psychtoolbox script [75]. The animals worked 5 days/week, 3 h/day, aiming to achieve about 200 correct trials each day.

Behavioral data consisting of stimulus and tap times were acquired using the same real‐time processor that collected the neural data. Subsequent data processing was performed using custom code in Matlab (Mathworks, Natick, MA). Statistical analysis was performed in R [76] using the *lme4* package [77].

### 2.3 Neural Recording

We used two high‐density electrode arrays developed at our lab [57] and equipped with the 64‐site Buzsaki64‐Z64 probe. The arrays were inserted semichronically in the medial areas of the premotor cortex (MPC), targeting SMA and pre‐SMA, one in the left hemisphere and one in the right hemisphere. The 128 channels were acquired, amplified, and digitized using a PZ2 preamplifier (Tucker‐Davis Technologies, Alachua, FL) at 24,414 Hz. The signal was transmitted to an RZ2 base station, and data were stored using an RS4 Data Streamer.

### 2.4 Spike Processing and Firing Rates

Unit activity was extracted from the continuous neural recordings using spike sorting algorithms. For M1, we used our custom sorting pipeline, ABVA [78]. For M2, we used KiloSort2 [79]. Spike sorting detects action potentials from the extracellular voltage signal and classifies them as different neurons based on their unique features, namely, shape, amplitude, and relative amplitude and relative timing across the spatially separated electrodes. A separate validation showed that spike patterns from ABVA were highly correlated with those from KiloSort2 [43].

A time series of neuronal firing rates was computed for each unit cell and each trial by convolving the spike series with a Gaussian kernel (*σ* = 10 ms, sampling rate of 1000 Hz). Trials from matching conditions on the same recording day were averaged. There were 402 cells in M1 and 1744 cells in M2 after rejecting units with a discharge rate lower than 2 spikes per second and signals not detected in all conditions.

### 2.5 Neural State Space Trajectories

The population activity was projected to a trajectory on a low‐dimensional manifold. This was done separately for each monkey using principal component analysis [59, 71]. The population activity **X** comprised the firing rates of *n* cells across *m* time points. At each point, population activity was a vector in an *n*‐dimensional embedding space. Linear combinations of dimensions of **X** were mapped to a new space **Y** = **XP** such that the new dimensions were uncorrelated and ranked by their variance. It has become customary to refer to these as principal components. The *n* × *n* projection matrix **P** was found using singular value decomposition. We retained the top principal components with a large proportion of the variability forming the low‐dimensional state space (see Section 2.6).

The projection matrix **P** was estimated globally, meaning that it was computed after pooling all neurons from all days. To avoid bias toward longer interval trials that contained more data, each trial was time‐normalized and resampled with interpolation to 50 samples per ISI. This means that time was expressed in stimulus cycles. Pooling the full population of units recorded across trials allowed us to infer the underlying attentional and stimulus synchronizing state on individual trials. Furthermore, this trial‐wise approach promoted the analysis of the neural trajectory along the multiple stimulus and tapping cycles of the trial. For easier interpretation, trial time was shifted relative to the onset of tapping so that the stimulus before the first tap was at *t* = 0.

### 2.6 Analysis

We parameterized the manifold along the length of the trial by using a trial‐continuous or cycle‐by‐cycle approach. Parameters of the population trajectory were computed using the stimulus onset and the beginning of tapping times as reference points. We analyzed how features of this trajectory evolved along the length of the trial over successive stimulus intervals because we were interested in the role of attending to the stimulus and tapping along repeated stimuli. We analyzed separately the amplitude, phase, and degree to which the neural trajectory obeyed an oscillatory dynamic. We used linear effects models separately for each monkey and each variable to test for effects of stimulus cycle number, stimulus interval duration, and correct performance.

*Amplitude* dynamics was defined as the distance of the trajectory at a point in time from the global mean. The rate of change of the amplitude was measured by taking its slope with respect to time, separately in the attention and tapping phases of the trial.

*Phase* dynamics was measured cycle‐by‐cycle by finding peaks of activity in individual dimensions using a peak‐picking algorithm and determining their phase relative to the closest stimulus.

#### 2.6.1 Index of Oscillation

**FIGURE 3:**
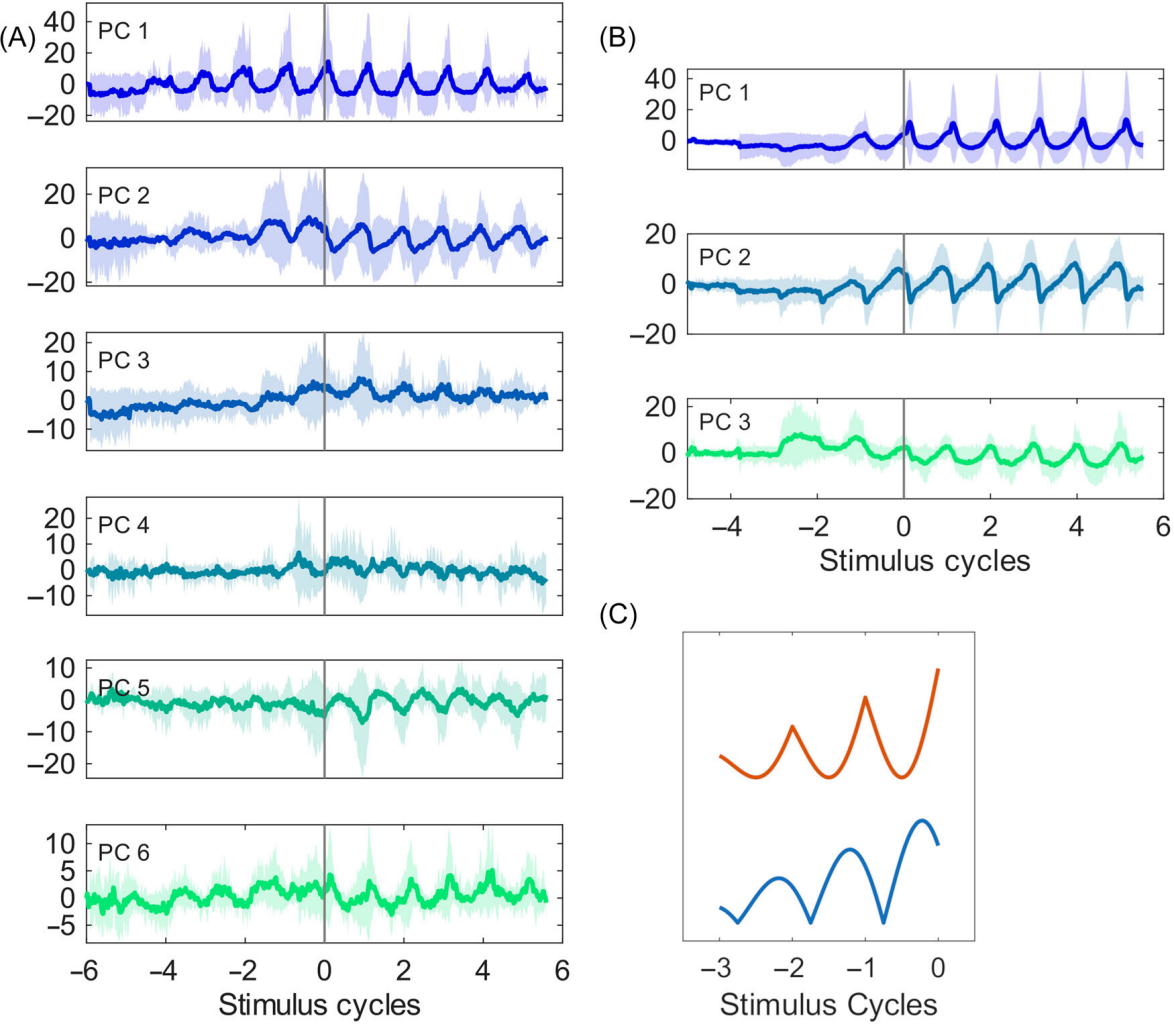
Individual dimensions of population dynamics. (A) Monkey 1 (correct trials, ISI = 850 ms, mean ± SD across sessions). (B) Monkey 2 (correct trials, ISI = 950 ms, mean ± SD across sessions). (A, B) Time is normalized to a unit stimulus. Stimulus = 0 marks the onset of tapping. (C) Two examples of the changing amplitude oscillation functions, which were used to fit the neural trajectory dynamics for each PC and compute the corresponding index of oscillation.

Preliminary observations suggested that each dimension of the population trajectory exhibited an asymmetric relaxation‐like nonlinear oscillation characterized by slow, smooth dynamics at the peak or valley and fast, cusp‐like dynamics at the opposite end. We accounted for the geometry of these oscillations, without modeling their generative dynamics, by fitting a function with the appropriate features. We defined an index of oscillation as the goodness‐of‐fit metric $R_{\mathrm{oscillation}}^{2}$ of a model with smooth oscillations and sharp peaks (see Figure 3C). The model, $\hat{y}=\left(a_{0}+a_{1}t\right)+\left(b_{0}+b_{1}t\right)\left(1-|\cos(\pi ft+\pi f/2-\theta /f)|\right)$, had free parameters for period, time‐varying baseline, time‐varying amplitude of the oscillation, and phase of its peaks relative to *t* = 0, the stimulus after which tapping started. It was fitted using nonlinear least squares optimization separately in the attention and tapping trial sections and in each principal component. After fitting the model, the $R_{\mathrm{oscillation}}^{2}$ was computed independently within each successive stimulus interval of the trial to get a time series of the index of oscillation for each stimulus interval along the length of the trial.

#### 2.6.2 Dimensionality

It is possible that the neural state space spans smooth high‐dimensional geometry instead of a low‐dimensional manifold. The former corresponds to a single scaling function that fits the full variance spectrum of the principal components [80]. We tested the hypothesis that a small number of top components, some of them with oscillatory dynamics, had higher eigenvalues than expected, indicating they diverged from the high‐dimensional geometry of background activity. We combined two techniques iteratively: regressing out a variance scaling power law from the principal component eigenvalues and clustering the residuals with *k*‐means to identify and remove outliers. This was inspired by a method for separating the periodic and aperiodic contributions to the EEG power spectrum [81].

We also considered nonlinear dimensionality reduction as suggested by recent literature [82, 83, 84]. We used nonlinear principal component analysis with a multilayer perceptron, which, like PCA, can project unseen data to the learned low‐dimensional space [85]. We did not observe qualitative differences in subsequent analyses. The interpretability of latent manifolds computed with nonlinear techniques can be difficult, yet their size is an informative parameter. We computed the intrinsic dimensionality of the manifold with a nonlinear method to serve as a lower boundary reference for the embedding dimensionality found with the linear method [61].

#### 2.6.3 Behavioral State Decoding

**FIGURE 4:**
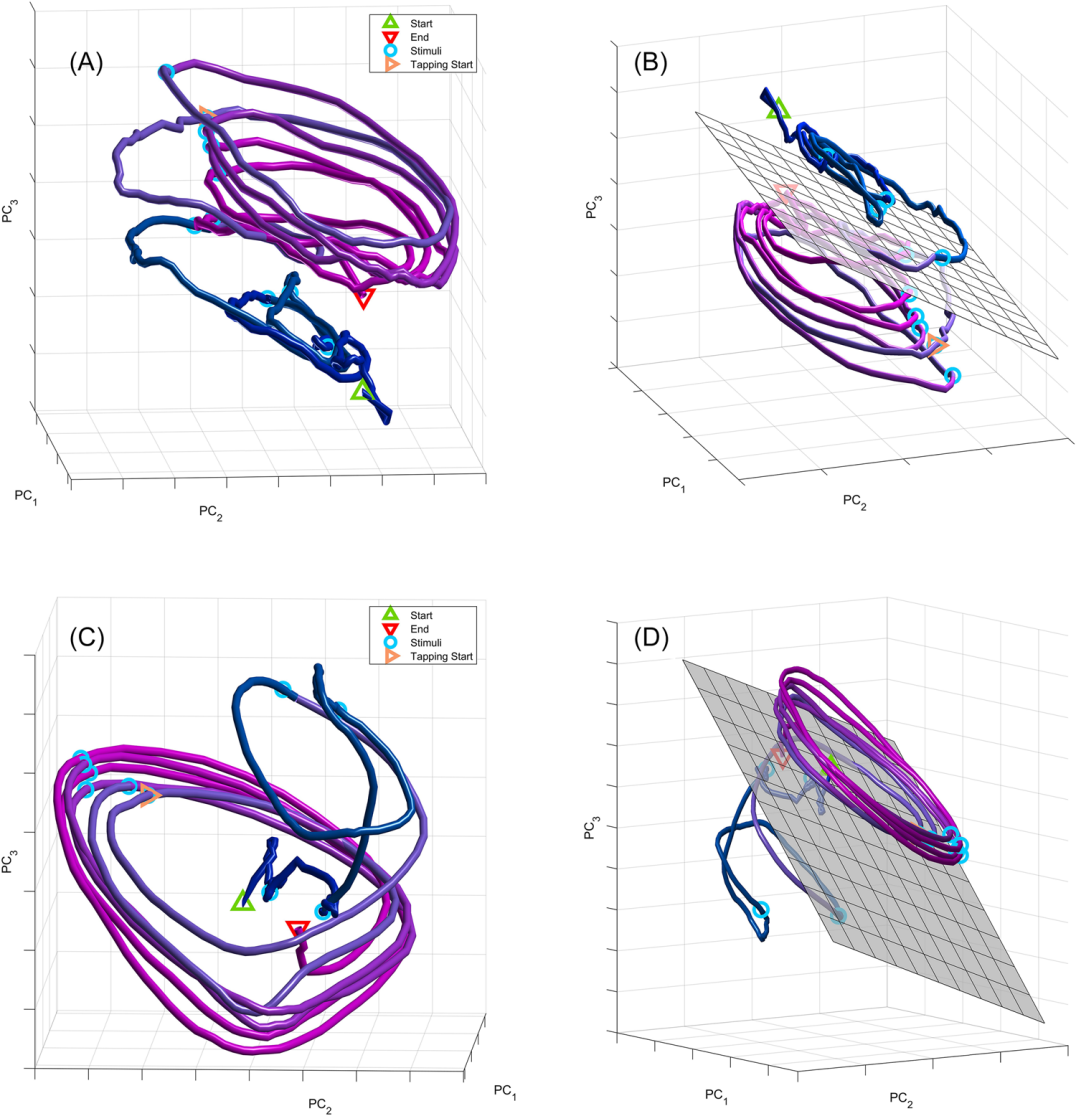
A) Population dynamics from Monkey 1 (correct trials, ISI = 850 ms). (B) The same dynamics of A are shown from a different perspective. A linear classifier plane was fitted to visualize the transition from attention to tapping. (C) Population dynamics from Monkey 2 (correct trials, ISI = 950 ms). (D) The same dynamics of C are shown from a different perspective. Again, a linear classifier plane was fitted to visualize the transition from attention to tapping.

We evaluated whether the behavioral states of attending and tapping could be classified reliably from the neural state. We trained a decoder on the top principal components selected with the dimensionality analysis. We used a support vector machine with a nonlinear polynomial kernel optimized on 80% of the data and tested on reserved 20% of the data. The decoder can be seen as a plane that separates the manifold into two regions (see Figure 4B,D). It classifies the behavioral state at a given point in time based on which side of the plane the trajectory is found at that point.

## 3 Results

### 3.1 Dimensionality

The size of the principal component subspace embedding the neural trajectory manifold was *d* = 6 in M1, explaining 36.6% of the population variance, and *d* = 3 in M2, explaining 43% of the variance. These dimensions were retained for subsequent analysis. Using a nonlinear approach, we found that the correlation dimension was *d* = 4.01 for M1 and *d* = 3.05 for M2. As expected, these are about the same or lower than the ones obtained from the PCA variance spectrum.

### 3.2 Amplitude

**FIGURE 5:**
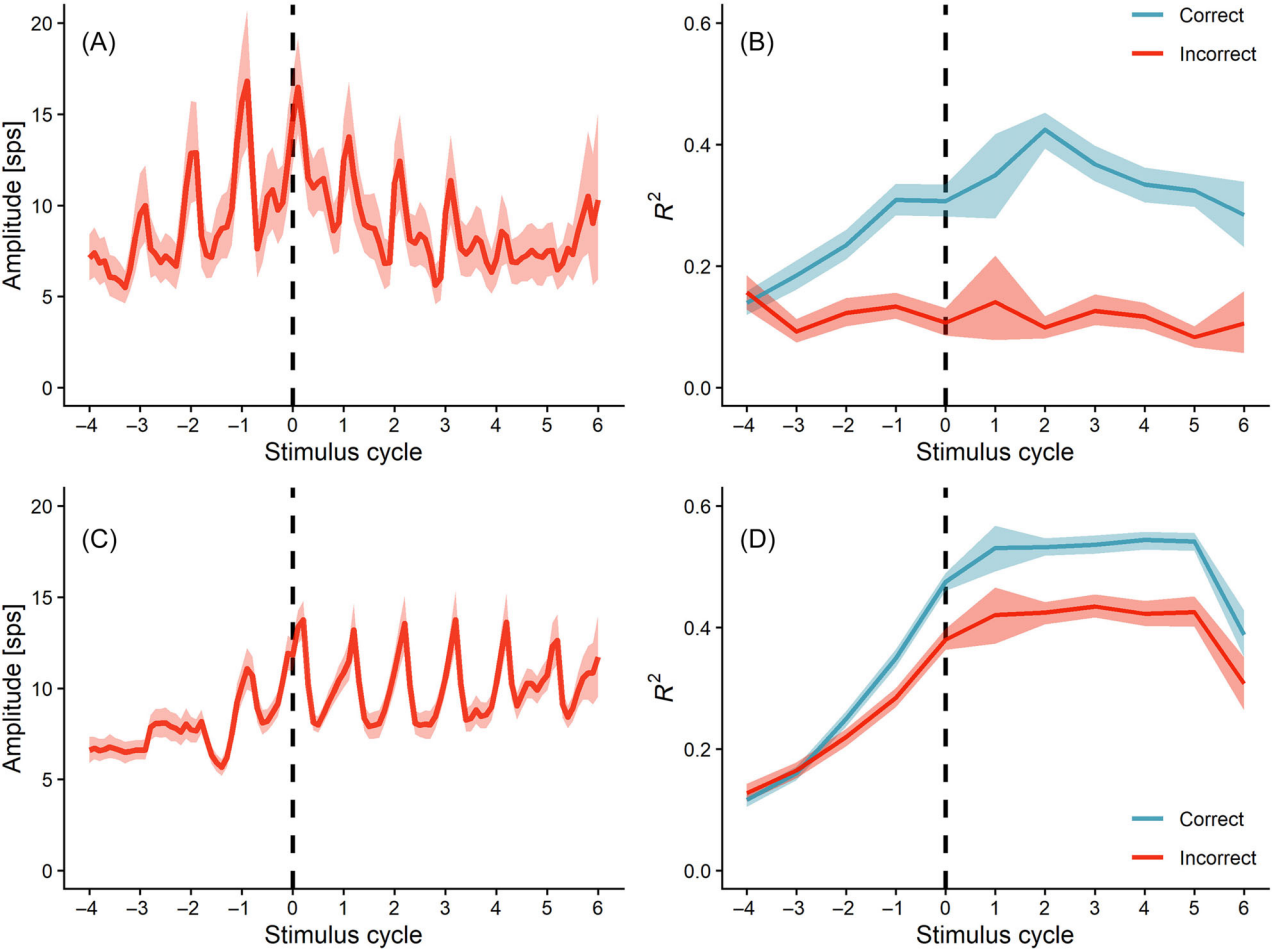
Dynamic parameters of the population trajectory. (A) Amplitude of the neural trajectory, averaged across recording sessions (Monkey 1, mean ± 95% CI, correct trials). sps stands for discharge rate. (B) The index of oscillation, averaged across manifold dimensions and sessions (Monkey 1, mean ± 95% CI), is shown separately for correct and incorrect trials. (C) Amplitude of the neural trajectory, averaged across recording sessions (Monkey 2, mean ± 95% CI, correct trials). (D) The index of oscillation, averaged across manifold dimensions and sessions (Monkey 2, mean ± 95% CI), is shown separately for correct and incorrect trials.

The time course of the neural trajectory demonstrated a pattern of rising amplitude oscillations during the attending epoch (see Figure 3 for individual dimensions and Figure 4 for the fully embedded trajectory). During the attending epoch of the trial, the population trajectory moved onto a spiral‐like trajectory with an increasing amplitude over successive stimulus intervals (see Figure 4). The rate of change of amplitude in that part of the trial was statistically higher than zero (*β* = 0.006, *t* = 3.71, *p* < 0.001 for M1 and *β* = 0.013, *t* = 6.27, *p* < 0.0001 for M2), as seen in Figure 5A,C. In both monkeys, the effects of ISI and all interactions were not significant (*p*s n.s.). Then the amplitude remained steady during the synchronization epoch (Figures 3A, B, 5A, and 5C), although the rate of change was negative (M1: *β* = −0.017, *t* = −3.93, *p* < 0.001; M2: *β* = −0.010, *t* = −2.57, *p* < 0.05). Larger amplitude was observed in both task epochs for correct than incorrect trials.

### 3.3 Index of Oscillation

Another form of evidence for the emergence of oscillatory dynamics was obtained by the time‐resolved index of oscillation, defined as the interval‐by‐interval goodness of fit of the oscillation function shown in Figure 3C. As can be seen in Figure 5B,D, the index increased during the attending part of the trial, peaked around the time of tapping onset, and was lower in incorrect trials. Indeed, the slope of the index with respect to successive stimulus cycles in the attending part was significantly greater than zero (M1: *β* = 0.023, *t* = 14.64, *p* < 0.001; M2: *β* = 0.074, *t* = 55.65, *p* < 0.001). In the tapping part of the trial, the slope with respect to successive stimulus cycles was negative (M1: *β* = −0.025, *t* = −6.69, *p* < 0.001; M2: *β* = −0.009, *t* = −4.55, *p* < 0.001). Importantly, the index of oscillation was lower in incorrect trials (M1: *β* = −0.262, *t* = −8.86, *p* < 0.001; M2: *β* = −0.118, *t* = −6.54, *p* < 0.001) and in higher principal components (M1: *β* = −0.021, *t* = −8.28, *p* < 0.001; M2: *β* = −0.042, *t* = −31.05, *p* < 0.001) (Figures 6B and 7B).

### 3.4 Phase

The phase of peaks of individual components relative to the stimuli showed little dependence on trial conditions. In the attending epoch of the trial, phase tended to increase with successive stimuli (M1: *β* = 0.017, *t* = 4.18, *p* < 0.001; M2: *β* = 0.054, *t* = 16.31, *p* < 0.001), and there were no significant associations with the other conditions (all *p*s n.s.). In the tapping part of the trial, phase also tended to increase (M1: *β* = 0.030, *t* = 4.46, *p* < 0.001; M2: *β* = 0.026, *t* = 4.96, *p* < 0.001). It was higher in incorrect trials in M2 (*β* = 0.047, *t* = 5.73, *p* < 0.001). This was not a significant effect in M1 (*β* = 0.013, *t* = 1.20, *p* = 0.23).

### 3.5 Decoding

As Figure 4B,D shows, the population trajectory moved into a different region as the trial shifted from attending to tapping. This is consistent with the idea that neural state spaces have different subspaces for different aspects of a task. To confirm this, the classifier was able to distinguish between the attending and tapping parts of the trial solely based on the selected top dimensions. The accuracy/precision/recall were 89%/92%/88% for M1 and 92%/90%/96% for M2, respectively.

## 4 Discussion

In this study, we recorded high‐density MPC neural activities during a rhythmic synchronization task performed by macaques. We found that the population dynamic was consistent with a low‐dimensional neural state trajectory on a manifold with interesting properties. The critical scientific observation of the present study is the emergence of elliptic rhythmic task‐related dynamics during the perception epoch, which is then consolidated in a different subspace during tapping synchronization. The neural population trajectories during the latter had an increase in amplitude and oscillatory coherence, presumably driven by the animals’ engagement in sequential rhythmic tapping. Previous studies in the macaque have shown rhythmic activity modulation with successive stimulus intervals in populations of cells in nuclei of the basal ganglia and cerebellum [86, 87]. Here, we observed that in the transition from attending to tapping, the trajectory reliably switched into a different state space region with a stable amplitude. Dynamic parameters, particularly the index of oscillation, were positively associated with correct trial performance during this epoch. Thus, our findings contribute to existing evidence that the geometry of low‐dimensional manifolds serves to implement task‐related computations such as attending to a metronome, extracting its tempo, and then synchronizing the tapping to this signal [66, 88, 89, 90, 91, 92].

The two monkeys exhibited consistent dynamic patterns. Yet, M1, which had overall poorer behavioral performance, also exhibited visibly lower index of oscillation than M2 in incorrect trials and lower accuracy of trial phase classification. Arguably, the index of oscillation, defined at the level of population dynamics, reflects the synchronization of the underlying neuronal substrate. As such, the results suggest that the organization of neuronal units in a coherent population dynamic is associated with better time keeping or with better focus on the task. In the same context, it is worth considering the possible pattern of results in an auditory task, given that the macaque is known to be adapted for vision‐based rather than hearing‐based coordination and has a less developed auditory–parietal connection. Consistent with this, the previous study from our lab found that geometrically comparable auditory and visual timing dynamics shared the space of the MPC during the tapping phase, but, as expected, the behavioral and neural population dynamic measures were lower in the auditory versus the visual conditions of performance [43].

Beyond their role in timing, the geometry of neural manifolds is linked to various behavioral and cognitive processes. For example, in the prefrontal cortex of nonhuman primates, decisions are represented by attractor basins, where the depth and steepness of these basins correlate with decision consistency and confidence [93]. Within the same region, a low‐dimensional neural embedding tracks the physical properties of an object, serving as a neural substrate for mental simulation during a physical reasoning task [94]. In the hypothalamus of mice, a neural line attractor encodes the intensity of aggressive states related to specific actions, such as sniffing and dominance mounting [95, 96]. Recent studies also demonstrate that neural manifolds can evolve within distinct planes corresponding to different task properties. For example, in the motor cortex, neural trajectories exhibit rotational dynamics to produce reach movements that unfold within different planes corresponding to different task conditions [97]. Similarly, in the human hippocampus, learning to perform an inference task produced the emergence of an abstract and disentangled representation of task variables on a neural manifold, with the context of the task encoded orthogonally to the stimulus [98]. All these results highlight the importance of the geometry of neural manifolds in a multitude of behaviors and brain regions.

How do these results inform the dynamic mechanisms involved in initiating and sustaining coordination with a rhythmic stimulus? We identified three nonmutually exclusive perspectives on the generation of rhythm‐tracking population dynamics. It is debatable whether large‐scale oscillations arising in response to a rhythmic stimulus result from the entrainment of endogenous oscillations or from a stimulus‐evoked transition between different dynamic regimes [99]. The gradual increase in amplitude and index of oscillation reported here is consistent with resonance, more so than entrainment, yet we cannot rule out that either of these is involved at some level in the generation of the neural trajectory.

We take advantage of these considerations to re‐focus the discussion on questions pertaining to the comparative abilities of humans and other animals. As reviewed above, macaques are capable of complex rhythmic perception and production skills. Yet, the animals perform such tasks for short trials, rarely exceeding 10 consecutive stimulus/tap cycles. Is this related to the dynamic nature of the neural trajectories reported here? The weaving, spiral‐like shape is a nonlinear property, and nonlinear dynamic systems are inherently poised to produce self‐sustained oscillations. The possibility of such oscillations is an intriguing but potentially paradoxical idea. A self‐sustaining oscillator in the MPC state space may mean that an animal has trouble exiting that subspace to stop the behavior and initiate a new behavior. In humans, in some circumstances, self‐sustained oscillations of brain dynamics can be maladaptive dynamic regimes associated with pathological states in humans, such as epileptic seizures [100], Parkinsonian tremor and freezing [101], and schizophrenia [102]. The trajectories reconstructed in the macaque MPC appeared to be nontangled, meaning that they do not recur in a strict sense, although testing this was not our objective here. This would suggest that MPC neural trajectories enabling the macaque's rhythmic tapping are not oscillators in a classical sense. Instead, the entire trial was driving the neural trajectory along a nonrepeating, nontangled path through state space where the sequence of cycles encoded the order of stimuli.

Another way of asking whether the population dynamics were self‐sustaining is by considering whether it was exclusively motor and stimulus driven [103]. Here, it did not appear to be motor‐driven because it started to increase during the attention phase of the trial. Yet, it also returned toward the initial state as soon as tapping stopped (see Figure 4). Hence, ambiguity remains about whether the observed dynamics in the macaque MPC can sustain a task‐related rhythm that can unfold indefinitely without ongoing stimulation. Nevertheless, in a previous study, we found that the circular neural population trajectories can be maintained for three internally driven continuation intervals during the classical synchronization continuation task [51, 74].

### 4.1 Oscillatory Bursting Activity Is Involved in Coordinating Individual Neurons

In the future, theoretical models need to address how individual neurons affect or define the dynamics in low‐dimensional manifolds linked to a task. In doing so, models need to reconcile the fact that the fine‐scale neurophysiology of brain oscillations is distinct from the smooth dynamics observed at the population scale. Cortical network oscillations constitute synchronized changes in excitability levels of neuronal circuits, with frequencies spanning from 0.01 to more than 200 Hz [104]. The local field potentials (LFPs) are voltage recordings that reflect the cumulative electrical activity of populations of neurons within 250 microns of the tip of an extracellular recording electrode. The oscillatory power of LFPs directly impacts the phase and probability of an individual neuron's discharge time, and the discharges of functionally related neurons are grouped during the excitatory phase of distinct oscillations. Neural assemblies grouped by oscillations can discharge to downstream targets, which require multiple active presynaptic synchronic inputs to produce an action potential [105].

Oscillations provide windows of opportunity in which neurons are sensitive to inputs via fluctuations between hyperpolarized and depolarized phases of neuronal membrane potentials. The phase of waves of activity can be coordinated with external stimuli [104] or with other internal neural processes to achieve large‐scale integration of information [106, 107, 108]. In this way, distinct populations of neurons can be integrated into transient task‐driven assemblies [109] for initiating motor activity [110, 111] or attentional and memory processes [31, 112]. In summary, coordinated oscillations enable information‐coding ensembles to effectively communicate across short and long spatial ranges and with systematic timeframes [113]. Yet, there are no self‐sustained smooth oscillations in the LFP. Instead, signals are composed of transient bursts, and action potentials travel within bursts of LFP oscillations [114, 115, 116]. Hence, it is evident that more work is needed to bridge the gap between bursting local oscillations, discharge rate information of single cells, and neural population manifolds as substrates of beat extraction and production. Various large‐scale, low‐dimensional theoretical models have been proposed to explain how collective dynamics organize neural ensembles into coherent functional units at different levels of neurophysiological detail [117]. Consequently, neural manifolds can inform such models and provide insight into what neural dynamics would be necessary for human‐like self‐sustained beat perception and production that unfold over long periods of time.

## Author Contributions

D.D.: design, data analysis, writing. A.B.: design, data collection, data analysis, paper edition. J.G.: design, data collection, data analysis, paper edition. H.M.: design, data analysis, writing.

## Conflicts of Interest

The authors declare no conflicts of interest.
